# Neutrophil gelatinase-associated lipocalin levels are U-shaped in the Ludwigshafen Risk and Cardiovascular Health (LURIC) study—Impact for mortality

**DOI:** 10.1371/journal.pone.0171574

**Published:** 2017-02-16

**Authors:** Rainer P. Woitas, Hubert Scharnagl, Marcus E. Kleber, Graciela E. Delgado, Tanja B. Grammer, Martin Pichler, Bernhard K. Krämer, Winfried März, Tatjana Stojakovic

**Affiliations:** 1 The Department of Internal Medicine I–Division of Nephrology, University of Bonn, Bonn, Germany; 2 Clinical Institute of Medical and Chemical Laboratory Diagnostics, Medical University of Graz, Graz, Austria; 3 Medical Clinic V (Nephrology, Hypertensiology, Endocrinology, Diabetology, Rheumatology), Medical Faculty Mannheim, University of Heidelberg, Mannheim, Germany; 4 Department of Internal Medicine, Division of Clinical Oncology, Medical University of Graz, Graz, Austria; 5 Synlab Academy, Synlab Services GmbH, Mannheim, Germany; University of Bologna, ITALY

## Abstract

**Introduction:**

Neutrophil gelatinase–associated lipocalin (NGAL) is a glycoprotein released by damaged renal tubular cells and mature neutrophils. It is elevated in kidney injury, but also in patients with coronary artery disease (CAD) and myocardial infarction. We investigated the prognostic value of NGAL for total and cardiovascular mortality in patients undergoing coronary angiography without history of renal insufficiency at inclusion into the study.

**Participants:**

The LURIC study is an ongoing prospective cohort study of patients referred for coronary angiography and is designed to evaluate determinants of cardiovascular health.

**Results:**

NGAL was determined in plasma of 2997 persons (mean age: 62.7 years; 69.7% men) with a follow up for 10 years. 2358 patients suffered from CAD and 638 did not–these patients served as controls. Stable CAD was found in 1408 and unstable CAD in 950 patients. Death rate from cardiovascular events and all causes was highest in patients within the 4^th^ quartile of NGAL (≥56 ng/ml, p<0.001 vs third quartile), even after adjustment for age and gender. According to multivariable-adjusted Cox analysis adjusting for well-known cardiovascular risk factors, as well as lipid lowering therapy, angiographic CAD, and C-reactive protein we found patients in the highest NGAL quartile being at increased risk for cardiovascular (hazard ratio (HR) 1.33, 95%CI 1.05–1.67, p = 0.016) and all cause mortality (HR 1.29 95%CI 1.07–1.55, p = 0.007) compared to those in the third quartile. The lowest risk was seen in the third quartile of NGAL (41–56 ng/ml) suggesting a U-shaped relationship between NGAL and mortality. Further adjustment for creatinine abrogated the predictive effect of NGAL. However, the 3^rd^ and 4^th^ quartiles of NGAL were significantly associated with higher neutrophil counts, which were associated with CAD, non-ST elevation and ST-elevation myocardial infarction (p<0.05).

**Conclusions:**

Plasma NGAL concentrations are mainly derived from neutrophils and do not predict mortality independent of renal function.

## Introduction

Neutrophil gelatinase-associated lipocalin (NGAL) is a 25 kDa glycoprotein of the lipocalin family, which consists of a single disulfide bridged polypeptide chain of 178 amino acid residues that is stored in granules of neutrophil leukocytes [1]. Although the major source of plasma NGAL are neutrophils it is also found in monocytes, hepatocytes, endothelial and smooth muscle cells, renal tubular cells [2, 3]. It has been linked to acute tubular kidney injury, chronic kidney disease progression, neutrophil activation and atherogenesis [4].

The most important biological functions of NGAL are the transport of small, hydrophobic ligands thus mediating inflammatory response and inhibition of bacterial growth [5, 6]. Inflammatory activity is mediated through binding to chemotactic peptides, leukotrienes, and platelet-activating factor [7, 8]. NGAL is upregulated in endothelial dysfunction and inflammatory vascular damage [9–11]. Elevated NGAL expression was also noted in atherosclerotic plaques and was associated with infiltrating inflammatory cells, thrombus formation, plaque hemorrhage, and central necrosis [3, 10, 12]. Furthermore, aldosterone induces metalloproteinase-9 (MMP-9) and MMP-9/NGAL protein complex in neutrophils [13]. Formation of this complex with MMP-9, which is an important mediator of plaque instability and vascular remodeling, may be involved in plaque rupture.

Circulating NGAL may indirectly contribute to inflammatory processes, atherogenesis and subsequent progression of cardiovascular diseases [7, 9, 14]. NGAL was an independent predictor of major adverse cardiovascular events and mortality [15–17]. Elevated NGAL levels were found in the presence of CAD and correlated with the severity of cardiovascular disease (CVD) [11]. Elevated NGAL was also found in patients with acute myocardial infarction (MI), being a strong outcome predictor in patients with ST–elevation MI (STEMI) [18]. Recently a strong association of NGAL with inflammation and with 10-year outcomes in the general population was noted. Furthermore, NGAL improved cardiovascular risk stratification when added to the Framingham risk score [19]. Therefore, we investigated the predictive role of NGAL for total and cardiovascular mortality in patients undergoing coronary angiography in the LURIC cohort [20].

## Material and methods

### Study design and participants

The Ludwigshafen Risk and Cardiovascular Health (LURIC) study is an ongoing prospective cohort study of patients referred for coronary angiography and is designed to evaluate the effects of biomarkers on the cardiovascular system. Study design and baseline examinations have been described previously in detail. [20]. In total, 3,316 subjects (2,309 men and 1,007 women) aged 18 to 95 years were recruited from July 1997 to January 2000 at the Heart Center Ludwigshafen in Germany. Patients were included into the study if a coronary angiogram was performed and clinical conditions were stable with the exception of an acute coronary syndrome (ACS). In 2997 patients plasma NGAL measurements were available. No study participants were lost to follow-up. We did not record how many subjects were suitable to inclusion, but who did not give written informed consent.

Patients, who suffered from any other diseases than ACS, e.g., chronic renal failure, severe rheumatic arthritis, persistent incapacitation or a history of malignancy were excluded.

Written informed consent was obtained from each participant. The study complies with the Declaration of Helsinki and was approved by the institutional review board at the Medical Association of Rheinland-Pfalz. Detailed descriptions of the LURIC baseline examination are provided elsewhere [20].

Brachial artery pressure values, the extent of CAD, and the severity of heart failure (HF) was assessed as described in detail previously [21]. The diagnosis of an ACS was establised when patients presented within 15 days of the beginning of disorders of unstable angina pectoris or acute MI, comprising non-ST-elevation MI (troponin T > 0.1 μg/L) and ST-elevation MI. Dyslipidaemia was defined as HDL cholesterol <1 mmol/L (40 mg/dL), and/or LDL cholesterol > 4.1 mmol/L (160 mg/dL), and/or triglycerides (TG) >2.4 mmol/L (200 mg/dL) [22]. Diagnosis of diabetes was established according to the definition of the American Diabetes Association [23]. Antihypertensive treatment [ACE-inhibitors (ACEi), Ang-II type-1 receptor blockers (ARBs), beta-blockers, calcium channel blockers, and diuretics] as well as the current smoking status were recorded accordingly.

The second follow-up of the LURIC cohort for mortality is the basis of the underlying analysis; the censoring date was June 30, 2009. During the follow-up no patients were lost. Causes of death were extracted from death certificates. Death certificates were missing for 18 deceased participants of the 2997 individuals who were included in the analysis for total mortality but excluded from the analysis of cardiovascular mortality.

### Laboratory parameters

Blood samples were drawn in the morning before cardiac catheterization, after subjects had fasted. Serum and EDTA plasma aliquots were shock frozen and stored at −80°C for later use. Leukocytes were prepared using a whole blood lyse no-wash method, according to manufacturer recommendations (BectonDickinson). Preparations were analyzed on a four-color flow cytometer (FACSCalibur, BectoneDickinson) by standard procedures [24].

Phlebotomy for venous blood samples was done in the morning before coronary angiography with the participants in supine position for 5–10 min before phlebotomy. Routine laboratory parameters were immediately measured whereas remaining blood samples were snap frozen for further determination and stored at -80°C until analysis. The standard laboratory methods have been described in detail elsewhere [20].

IDMS-traceable creatinine was determined by liquid chromatography tandem mass spectrometry (LC-MS/MS) [25]. Within-day and between-day coefficients of variation (CVs) were 4.5% (59 μmol/L) and 2.9% (477 μmol/L), and 9.6% (53 μmol/L) and 7.5% (504 μmol/L), respectively. High-sensitive (hs) C-reactive protein (CRP) and cystatin C were measured by immunonephelometry (N-High-Sensitive CRP; N-Latex cystatin C, Dade Behring, Marburg, Germany) using a Behring nephelometer II. The intra-assay and inter-assay CVs were 2.2% and 2.5%, respectively as described in detail previously [21]. The CKD Epidemiology Collaboration (CKD-EPI) equation (eGFR_CKD-EPI_) was used to calculate estimated glomerular filtration rate (eGFR) [26]. Neutrophil gelatinase-associated lipocalin (NGAL) was measured in EDTA-plasma using a particle-enhanced turbidimetric immunoassay (BioPorto Diagnostics, Gentofte, Denmark) on an Olympus AU640 analyzer (Olympus Diagnostika, Hamburg, Germany). The day-to-day coefficient of variation was <3%.

### Statistical analysis

Continuous parameters following non-normal distributions underwent natural logarithmic transformation. Baseline characteristics are given as percentages for categorical data, and depending on their distribution, continuous data are presented as mean±SD (normal distribution) or as geometric means with 95% confidence intervals (95%CI) (skewed distribution). Comparisons between groups were performed by analysis of variance for continuous parameters and by Pearson's chi-square test for categorical variables. We studied the effects of gender, age, CAD, and cardiovascular risk factors, on NGAL using ANOVA models in which we included those factors not under examination as co-variables; NGAL values were logarithmically transformed prior to statistical analysis. Kaplan-Meier survival function with the log-rank test for equality was used to evaluate the predictive ability of quartiles of NGAL or neutrophils with overall and CVD mortality.

Time-to-event analyses were performed using multivariate Cox proportional-hazards regression. Established cardiovascular risk factors were determined a priori as co-variables for multivariate adjustments. Besides the crude and unadjusted model, model 2 was adjusted for age (continuous variable) and gender, model 3 was adjusted in addition to age and sex for coronary artery disease (none, stable CAD, unstable CAD, NSTEMI, STEMI), body mass index (continuous variable), diabetes mellitus (coded as binary variable), hypertension (coded as binary variable), smoking (coded as never, previous or current), HDL- and LDL-cholesterol, triglycerides (all used as continuous variables), lipid lowering therapy (coded as binary variable). Model 4 was additionally adjusted for hs CRP (continuous variable), and model 5 for creatinine.

Analyses were considered significant at a 2-tailed p-value of <0.05. All calculations were performed using SPSS Version 21.0.

## Results

### Associations of NGAL with cardiovascular risk factors and CAD

Clinical and biochemical characteristics of the study participants with and without angiographic established CAD are shown in S1 Table. Patients with CAD had higher cystatin C and creatinine levels and consequently a somewhat higher NGAL plasma level (not significant). As expected, patients with CAD were older, male, suffered from insulin resistance or manifest diabetes and hypertension and had a history of smoking. Consequently, dyslipidemia and vascular complications were more frequent than in individuals without CAD (S1 Table).

No differences in plasma NGAL levels were noted between male and females. However, NGAL levels increased age-dependently and were 8.7 and 12.4% higher in persons older than 60 and 70 years, respectively, as compared to those younger than 60 years (Table 1).

**Table 1:** Association of NGAL with cardiovascular risk factors and coronary artery disease.

	n	NGAL (ng/mL)^a^	Difference (%)^b^	*P*^c^
**Gender**				
Men	2069	40.3 (39.2–41.5)		
Women	927	40.0 (38.2–41.8)	-0,7	0.759
**Age, years**				
<60	1098	37.8 (36.3–39.4)		
60–70	1071	41.1 (39.5–42.7)	+8.7	0.005
>70	827	42.5 (40.5–44.5)	+12.4	<0.001
**Coronary artery disease**				
None	638	40.0 (38.0–42.3)		
Stable CAD	1408	41.1 (39.8–42.6)	+2.8	0.415
Unstable CAD (Troponin T-)	585	39.3 (37.2–41.4)	-1,8	0.606
NSTEMI or STEMI (Troponin T+)	365	38.5 (35.9–41.2)	-3,8	0.393
**Body mass index, kg/m**^**2**^				
≤27 or 26^d^	1407	41.9 (40.4–43.4)		
>27 or 26^d^	1589	38.8 (37.6–40.1)	-7,4	0.002
**Waist circumference, cm**				
≤102 or 88^e^	1564	41.7 (40.3–43.1)		
>102 or 88^e^	1432	38.6 (37.3–40.0)	-7,4	0.003
**Diabetes mellitus**				
No	1810	41.5 (40.2–42.8)		
Yes	1186	38.3 (36.9–39.8)	-7,7	0.002
**Insulin resistance by HOMA**				
≤2.5	1703	41.2 (39.9–42.6)		
>2.5	1209	38.8 (37.3–40.3)	-5,8	0.023
**Hypertension**				
No	815	39.8 (38.0–41.7)		
Yes	2181	40.4 (39.3–41.5)	+1.5	0.631
**Smoking**				
Never	1087	40.2 (38.6–41.9)		
Former	1341	40.2 (38.8–41.7)	0	0.996
Current	568	40.2 (37.9–42.6)	0	0.975
**Lipid-lowering drugs**				
No	1530	40.9 (39.5–42.3)		
Yes	1466	39.5 (38.2–40.9)	-3,4	0.184
**LDL cholesterol, g/L**				
1^st^ quartile (<0.95)	758	39.4 (37.6–41.3)		
2^nd^ quartile (0.95–1.14)	746	39.7 (37.9–41.6)	+0.8	0.844
3^rd^ quartile (1.15–1.38)	755	39.6 (37.8–41.5)	+0.5	0.886
4^th^ quartile (≥1.39)	737	42.3 (40.3–44.3)	+7.4	0.040
**HDL cholesterol, g/L**				
1^st^ quartile (<0.32)	761	43.5 (41.4–45.7)		
2^nd^ quartile (0.32–0.37)	762	40.3 (38.5–42.2)	-7,4	0.023
3^rd^ quartile (0.38–0.44)	692	38.5 (36.8–40.6)	-11,5	0.001
4^th^ quartile (≥0.45)	781	38.5 (36.7–40.4)	-11,5	0.001
**Triglycerides, g/L**				
1^st^ quartile (<1.09)	749	40.0 (38.1–42.1)		
2^nd^ quartile (1.09–1.46)	746	40.5 (38.7–42.5)	+1.3	0.729
3^rd^ quartile (1.47–2.00)	751	41.0 (39.1–42.9)	+2.5	0.499
4^th^ quartile (≥2.01)	750	39.3 (37.4–41.2)	-1,8	0.594
**C-reactive protein, mg/L**				
<3	1408	38.3 (37.0–39.7)		
3–10	979	41.4 (39.8–43.2)	+8.1	0.005
≥10	609	42.9 (40.6–45.3)	+12.0	0.001
**eGFR, mL/min/1.73m**^**2**^ **(CKD-EPI)**				
>90	1093	32.4 (31.1–33.7)		
61–90	1482	40.2 (39.0–41.5)	+24.1	<0.001
≤60	418	70.0 (65.8–74.5)	+116.0	<0.001
**Creatinine, mg/dL**				
1^st^ quartile (<0.72)	670	32.2 (30.6–33.9)		
2^nd^ quartile (0.72–0.85)	714	37.0 (35.4–38.7)	+14.9	<0.001
3^rd^ quartile (0.86–0.99)	698	40.9 (39.1–42.9)	+27.0	<0.001
4^th^ quartile (≥1.00)	692	53.9 (51.4–56.5)	+64.7	<0.001
**Cystatin C, mg/L**				
1^st^ quartile (<0.81)	744	32.1 (30.6–33.6)		
2^nd^ quartile (0.81–0.91)	756	35.6 (34.0–37.2)	+10.9	0.002
3^rd^ quartile (0.92–1.06)	738	40.6 (38.8–42.5)	+26.5	<0.001
4^th^ quartile (≥1.07)	758	56.3 (53.7–59.0)	+75.4	<0.001
**Friesinger score**				
1^st^ quartile	605	38.3 (35.9–40.9)		
2^nd^ quartile	698	42.1 (40.1–44.2)	+9.9	0.022
3^rd^ quartile	977	40.0 (38.4–41.8)	+4.4	0.314
4^th^ quartile	716	40.3 (38.3–42.4)	+5.2	0.282

^a^Estimated marginal means and 95% confidence intervals obtained in a general linear model (ANOVA), adjusted for sex, gender, coronary artery disease, body mass index, diabetes mellitus, hypertension, smoking, LDL cholesterol, HDL cholesterol, triglycerides

^b^Compared to the first category of each variable

^c^Post hoc pairwise comparisons with the first category of each variable

^d^Thresholds of 27 and 26 kg apply to males and females, respectively

^e^Thresholds of 102 and 88 cm apply to males and females, respectively

Patients with stable CAD had slightly higher NGAL concentrations than individuals without CAD, but those with unstable CAD, NSTEMI, or STEMI had slightly lower NGAL concentrations (not significant). Body mass index, waist circumference, diabetes mellitus and insulin resistance were inversely associated with NGAL concentrations (Table 1). When we looked at the NGAL levels in patients with and without CAD separately, the associations in individuals without CAD were abolished (S2 Table).

Smoking, dyslipidemia, uric acid, triglycerides, prevalence of peripheral vascular disease, blood pressure, use of antiplatelet and lipid lowering drugs, AT1 receptor antagonists as well as calcium channel blockers were not related to NGAL (data not shown). However, HDL cholesterol was associated with lower NGAL. In contrast to this, only the 4^th^ quartile of LDL cholesterol was associated with a small, but significant NGAL increase (7.4% compared to the 1^st^ quartile). CRP was positively associated with NGAL concentrations.

eGFR was inversely related to NGAL and vice versa; likewise, NGAL increased significantly in parallel with creatinine and cystatin C which was seen in patients with CAD and individuals without CAD (S2 Table). Finally, the 2^nd^ quartile of the Friesinger score showed the highest concentration of NGAL (p = 0.022), whereas values were lower in the 3^rd^ and 4^th^ quartile.

### Clinical and biochemical patient characteristics according to quartiles of NGAL

Patients in the 4^th^ quartile of NGAL were at significantly higher age than the others, whereas no differences were noted with respect to sex (Table 2). Interestingly, the highest percentage of diabetes was noted in the first quartile of NGAL. Conversely, significant differences were seen for blood glucose, cholesterol, triglycerides, CRP, eGFR, creatinine, cystatin C (Table 2) as well as for the use of ACE inhibitors, diuretics, antibiotics, glucocorticoids (data not shown).

**Table 2:** Clinical and biochemical characteristics of study participants at baseline.

	NGAL, ng/mL				
	<30	30–40	41–56	≥56	*P*^a^
	(n = 728)	(n = 765)	(n = 775)	(n = 729)	
Age, years	62±11	62±11	62±10	65±10	<0.001
Male sex	70	68	69	70	0.740^b^
Body mass index, kg/m^2^	28±4	27±4	28±4	27±4	0.015
Waist circumference, cm	100±12	99±12	99±12	98±12	0.032
Diabetes mellitus %	47	36	35	41	<0.001
Insulin resistance by HOMA	3.5±4.4	3.1±3.4	3.1±3.6	3.1±4.1	0.061
Systemic hypertension %	74	69	74	75	0.065
Smoking %					
Never	36	36	37	36	
Past	44	46	45	44	
Current	20	18	18	19	0.964
Previous myocardial infarction %	42	38	40	45	0.023
Peripheral vascular disease %	9	9	9	11	0.599
Cerebrovascular disease %	9	7	9	11	0.049
Systolic blood pressure, mmHg	142±24	140±23	141±23	142±25	0.501^c^
Diastolic blood pressure, mmHg	81±11	81±11	81±11	80±12	0.107^c^
Fasting blood glucose, g/L	1.18±0.39	1.12±0.35	1.11±0.32	1.12±0.36	<0.001
LDL cholesterol, g/L	1.13±0.33	1.16±0.33	1.20±0.36	1.17±0.35	0.001^d^
HDL cholesterol, g/L	0.38±0.11	0.40±0.10	0.40±0.11	0.38±0.11	<0.001^d^
Triglycerides, g/L	1.54 (1.14–2.10)	1.37 (1.04–1.90)	1.46 (1.09–1.98)	1.52 (1.13–2.02)	<0.001^d^^,^^e^
C-reactive protein, mg/L	3.6 (1.4–9.3)	2.7 (1.1–6.8)	3.0 (1.1–7.2)	4.4 (1.7–10.7)	<0.001^d^^,^^e^
eGFR, mL/min (CKD-EPI)	88±17	87±17	82±16	69±23	<0.001
Creatinine, mg/dL	0.82±0.21	0.84±0.21	0.88±0.23	1.14±0.92	<0.001
Cystatin C, mg/L	0.90±0.20	0.90±0.19	0.96±0.23	1.24±0.69	<0.001

Values are mean±SD, % or median (25^th^-75^th^ percentile), respectively

^a^ANOVA or logistic regression, respectively, adjusted for age and gender

^b^Logistic regression, adjusted for age only

^c^Adjusted for use of beta blockers, ACE inhibitors, AT1 receptor antagonists, calcium channel blockers, diuretics and lipid-lowering drugs

^d^Adjusted for use of lipid-lowering drugs

^e^ANOVA of logarithmically transformed values

### NGAL and risk prediction in CAD

NGAL was available in 2997 persons (mean age: 62.7±10.5 years; 30.3% women) with a follow up for 10 years. Of those 2359 suffered from CAD and 638 did not. 900 patients died, 571 of those were due to cardiovascular causes (data not shown). CAD was clinically stable in 1408 and unstable (angina pectoris, NSTEMI, STEMI) in 951 patients, respectively.

All cause and cardiovascular mortality were highest in patients within the 4^th^ quartile of NGAL (hazard ratio, (HR) 1.36 95%CI 1.13–1.63 and HR 1.39 95%CI 1.11–1.75)), even after adjustment for age and gender (Tables 3 and 4).

**Table 3:** Hazard ratios (HR) for death from all causes according to NGAL.

NGAL (ng/mL)	Deaths	Model 1	*P*	Model 2	*P*	Model 3	*P*	Model 4	*P*	Model 5	*P*
	n (%)	HR (95% CI)^a^		HR (95% CI)^b^		HR (95% CI)^c^		HR (95% CI)^d^		HR (95% CI)^e^	
All individuals (n = 2997)											
1^st^ quartile (<30)	227 (31)	1.29 (1.06–1.56)	0.009	1.23 (1.01–1.49)	0.036	1.12 (0.92–1.36)	0.259	1.11 (0.92–1.35)	0.286	1.14 (0.93–1.39)	0.200
2^nd^ quartile (30–40)	195 (26)	1.01 (0.83–1.23)	0.936	1.00 (0.82–1.22)	0.999	0.99 (0.81–1.21)	0.935	0.99 (0.81–1.21)	0.905	0.98 (0.80–1.21)	0.863
3^rd^ quartile (41–56)	196 (25)	1.0^reference^		1.0^reference^		1.0^reference^		1.0^reference^		1.0^reference^	
4^th^ quartile (≥56)	282 (39)	1.68 (1.40–2.02)	<0.001	1.36 (1.13–1.63)	0.001	1.30 (1.08–1.56)	0.006	1.29 (1.07–1.55)	0.007	1.18 (0.97–1.43)	0.100

^a^ Model 1: unadjusted

^b^ Model 2: adjusted for age and gender

^c^ Model 3: additionally adjusted for coronary artery disease (none, stable CAD, unstable CAD, NSTEMI, STEMI), body mass index, diabetes mellitus, hypertension, smoking status, LDL cholesterol, HDL cholesterol, triglycerides

^d^ Model 4: additionally adjusted for C-reactive protein

^e^ Model 5: additionally adjusted for creatinine

**Table 4:** Hazard ratios (HR) for death from cardiovascular causes according to NGAL.

NGAL (ng/mL)	Deaths	Model 1	*P*	Model 2	*P*	Model 3	*P*	Model 4	*P*	Model 5	*P*
	n (%)	HR (95% CI)^a^		HR (95% CI)^b^		HR (95% CI)^c^		HR (95% CI)^d^		HR (95% CI)^e^	
All individuals (n = 2978)											
1^st^ quartile (<30)	141 (20)	1.26 (0.99–1.61)	0.059	1.20 (0.94–1.53)	0.137	1.07 (0.84–1.37)	0.585	1.06 (0.83–1.36)	0.626	1.11 (0.86–1.43)	0.437
2^nd^ quartile (30–40)	123 (16)	1.01 (0.78–1.29)	0.960	1.00 (0.78–1.28)	0.980	0.99 (0.77–1.27)	0.926	0.98 (0.77–1.26)	0.896	1.00 (0.77–1.30)	0.983
3^rd^ quartile (41–56)	124 (16)	1.0^reference^		1.0^reference^		1.0^reference^		1.0^reference^		1.0^reference^	
4^th^ quartile (≥56)	183 (25)	1.72 (1.37–2.16)	<0.001	1.39 (1.11–1.75)	0.005	1.34 (1.06–1.69)	0.013	1.33 (1.05–1.67)	0.016	1.24 (0.97–1.58)	0.091

^a^ Model 1: unadjusted

^b^ Model 2: adjusted for age and gender

^c^ Model 3: additionally adjusted for coronary artery disease (none, stable CAD, unstable CAD, NSTEMI, STEMI), body mass index, diabetes mellitus, hypertension, smoking status, LDL cholesterol, HDL cholesterol, triglycerides

^d^ Model 4: additionally adjusted for C-reactive protein

^e^ Model 5: additionally adjusted for creatinine

Multivariable-adjusted Cox analysis additionally adjusting for cardiovascular risk factors, lipid lowering therapy, angiographic coronary artery disease, and C-reactive protein demonstrated patients in the highest NGAL quartile being at increased risk for cardiovascular (HR1.34, 95%CI 1.06–1.69) and all cause mortality (HR 1.30 (95%CI 1.08–1.56) compared to those in the third quartile. Since the lowest risk was seen in the third quartile of NGAL (3^rd^ quartile), these data suggest an U-shaped relationship between NGAL and mortality which was true for all patients irrespective of whether they presented with stable CAD, unstable CAD, NSTEMI and STEMI (data not shown). Adjusting for creatinine abolished the significant associations of NGAL with mortality (model 5, Tables 3 and 4). We conducted a subgroup analysis according to an eGFR greater versus smaller (or equal) 60 ml/min/1.73m^2^. All cause and cardiovascular mortality rates were considerably higher (60.3% vs. 25.1% and 42.0 vs.15.5%, respectively) in those patients with low compared to higher eGFR (>60 ml/min/1.73m^2^) (Tables 5 and 6).

**Table 5:** Hazard ratios (HR) for death from all causes according to NGAL (eGFR >60 ml/min/1.73 m^2^).

NGAL (ng/mL)	Deaths	Model 1	*P*	Model 2	*P*	Model 3	*P*	Model 4	*P*	Model 5	*P*
	n (%)	HR (95% CI)^a^		HR (95% CI)^b^		HR (95% CI)^c^		HR (95% CI)^d^		HR (95% CI)^e^	
All individuals (n = 2576)											
1^st^ quartile (<30)	200 (29)	1.44 (1.17–1.79)	0.001	1.35 (1.09–1.67)	0.006	1.23 (0.99–1.52)	0.061	1.22 (0.98–1.51)	0.073	1.17 (0.94–1.47)	0.166
2^nd^ quartile (30–40)	166 (23)	1.10 (0.88–1.37)	0.396	1.11 (0.89–1.38)	0.379	1.10 (0.88–1.37)	0.420	1.09 (0.88–1.37)	0.430	1.05 (0.83–1.32)	0.705
3^rd^ quartile (41–56)	148 (21)	1.0^reference^		1.0^reference^		1.0^reference^		1.0^reference^		1.0^reference^	
4^th^ quartile (≥56)	133 (28)	1.34 (1.06–1.70)	0.013	1.22 (0.97–1.55)	0.094	1.19 (0.94–1.51)	0.141	1.18 (0.94–1.50)	0.162	1.10 (0.86–1.41)	0.459

^a^ Model 1: unadjusted

^b^ Model 2: adjusted for age and gender

^c^ Model 3: additionally adjusted for coronary artery disease (none, stable CAD, unstable CAD, NSTEMI, STEMI), body mass index, diabetes mellitus, hypertension, smoking status, LDL cholesterol, HDL cholesterol, triglycerides

^d^ Model 4: additionally adjusted for C-reactive protein

^e^ Model 5: additionally adjusted for creatinine

**Table 6:** Hazard ratios (HR) for death from cardiovascular causes according to NGAL (eGFR >60 ml/min/1.73 m^2^).

NGAL (ng/mL)	Deaths	Model 1	*P*	Model 2	*P*	Model 3	*P*	Model 4	*P*	Model 5	*P*
	n (%)	HR (95% CI)^a^		HR (95% CI)^b^		HR (95% CI)^c^		HR (95% CI)^d^		HR (95% CI)^e^	
All individuals (n = 2561)											
1^st^ quartile (<30)	128 (19)	1.53 (1.17–2.01)	0.002	1.43 (1.09–1.88)	0.009	1.28 (0.97–1.68)	0.080	1.26 (0.96–1.66)	0.097	1.24 (0.93–1.65)	0.148
2^nd^ quartile (30–40)	105 (15)	1.16 (0.87–1.54)	0.308	1.16 (0.88–1.54)	0.296	1.15 (0.86–1.52)	0.349	1.14 (0.86–1.52)	0.360	1.11 (0.83–1.49)	0.488
3^rd^ quartile (41–56)	89 (13)	1.0^reference^		1.0^reference^		1.0^reference^		1.0^reference^		1.0^reference^	
4^th^ quartile (≥56)	75 (16)	1.26 (0.92–1.71)	0.145	1.14 (0.84–1.55)	0.397	1.13 (0.83–1.54)	0.425	1.12 (0.82–1.53)	0.470	1.04 (0.75–1.44)	0.816

^a^ Model 1: unadjusted

^b^ Model 2: adjusted for age and gender

^c^ Model 3: additionally adjusted for coronary artery disease (none, stable CAD, unstable CAD, NSTEMI, STEMI), body mass index, diabetes mellitus, hypertension, smoking status, LDL cholesterol, HDL cholesterol, triglycerides

^d^ Model 4: additionally adjusted for C-reactive protein

^e^ Model 5: additionally adjusted for creatinine

Among patients with an eGFR > 60 ml/min/1.73m^2^ the highest number of deaths from all causes occurred in the first quartile even after adjustment for creatinine (HR 1.17 95%CI 0.94–1.47) for cardiovascular deaths a consistent trend was noted HR 1.24 95%CI 0.93–1.65). In the 418 patients with impaired renal function (eGFR ≤60 ml/min/1.73m^2^) no significant effects were seen (Tables 7 and 8). When we plotted eGFR, hsCRP and NGAL in a three-dimensional graph we found a rather weak association of CRP with NGAL in the eGFR range of 60–90 ml/min/1.73m^2^, but not in those subjects with good (higher than 90 ml/min/1.73m^2^) and reduced (less than sixty ml/min/1.73m^2^) kidney function (S1 Fig).

**Table 7:** Hazard ratios (HR) for death from all causes according to NGAL (eGFR ≤60 ml/min/1.73 m^2^).

NGAL (ng/mL)	Deaths	Model 1	*P*	Model 2	*P*	Model 3	*P*	Model 4	*P*	Model 5	*P*
	n (%)	HR (95% CI)^a^		HR (95% CI)^b^		HR (95% CI)^c^		HR (95% CI)^d^		HR (95% CI)^e^	
All individuals (n = 418)											
1^st^ quartile (<30)	26 (63)	1.24 (0.77–2.00)	0.372	1.15 (0.71–1.87)	0.558	1.06 (0.65–1.73)	0.815	1.06 (0.65–1.73)	0.815	1.24 (0.75–2.06)	0.404
2^nd^ quartile (30–40)	29 (58)	0.99 (0.62–1.57)	0.961	0.88 (0.56–1.41)	0.601	0.87 (0.54–1.38)	0.546	0.87 (0.54–1.38)	0.546	1.01 (0.62–1.64)	0.963
3^rd^ quartile (41–56)	48 (59)	1.0^reference^		1.0^reference^		1.0^reference^		1.0^reference^		1.0^reference^	
4^th^ quartile (≥56)	149 (61)	1.06 (0.76–1.46)	0.743	0.99 (0.72–1.38)	0.963	0.94 (0.68–1.31)	0.732	0.94 (0.68–1.31)	0.732	0.94 (0.66–1.33)	0.717

^a^ Model 1: unadjusted

^b^ Model 2: adjusted for age and gender

^c^ Model 3: additionally adjusted for coronary artery disease (none, stable CAD, unstable CAD, NSTEMI, STEMI), body mass index, diabetes mellitus, hypertension, smoking status, LDL cholesterol, HDL cholesterol, triglycerides

^d^ Model 4: additionally adjusted for C-reactive protein

^e^ Model 5: additionally adjusted for creatinine

**Table 8:** Hazard ratios (HR) for death from cardiovascular causes according to NGAL (eGFR ≤60 ml/min/1.73 m^2^).

NGAL (ng/mL)	Deaths	Model 1	*P*	Model 2	*P*	Model 3	*P*	Model 4	*P*	Model 5	*P*
	n (%)	HR (95% CI)^a^		HR (95% CI)^b^		HR (95% CI)^c^		HR (95% CI)^d^		HR (95% CI)^e^	
All individuals (n = 414)											
1^st^ quartile (<30)	13 (33)	0.85 (0.45–1.61)	0.624	0.80 (0.42–1.52)	0.499	0.70 (0.37–1.33)	0.277	0.70 (0.36–1.33)	0.275	0.80 (0.40–1.59)	0.525
2^nd^ quartile (30–40)	18 (36)	0.85 (0.48–1.49)	0.561	0.77 (0.43–1.36)	0.364	0.75 (0.42–1.33)	0.320	0.75 (0.42–1.34)	0.328	0.93 (0.51–1.70)	0.819
3^rd^ quartile (41–56)	35 (43)	1.0^reference^		1.0^reference^		1.0^reference^		1.0^reference^		1.0^reference^	
4^th^ quartile (≥56)	108 (44)	1.06 (0.72–1.55)	0.764	1.01 (0.69–1.48)	0.980	0.95 (0.65–1.41)	0.811	0.96 (0.65–1.41)	0.816	1.01 (0.66–1.53)	0.978

^a^ Model 1: unadjusted

^b^ Model 2: adjusted for age and gender

^c^ Model 3: additionally adjusted for coronary artery disease (none, stable CAD, unstable CAD, NSTEMI, STEMI), body mass index, diabetes mellitus, hypertension, smoking status, LDL cholesterol, HDL cholesterol, triglycerides

^d^ Model 4: additionally adjusted for C-reactive protein

^e^ Model 5: additionally adjusted for creatinine

As expected we found a significant association between NGAL and the absolute and relative neutrophil count (p<0.001, data not shown). The 3^rd^ and 4^th^ quartiles of NGAL were significantly associated with both, higher neutrophil count and higher percentage of neutrophils. Neutrophil counts were associated with CAD, non ST elevation and ST elevation myocardial infarction (NSTEMI and STEMI, p<0.05, data not shown).

## Discussion

In this study we investigated prospectively associations of plasma NGAL with all-cause and cardiovascular mortality in the LURIC cohort which represents an extremely well characterized population without overt kidney disease [20]. Even mildly impaired glomerular filtration, estimated by serum creatinine or serum cystatin C, or albuminuria predict cardiovascular and all-cause mortality. Consequently, chronic renal impairment is now considered a significant cardiovascular risk factor [27–29].

Our most important finding is that NGAL was independently associated with all-cause and CVD mortality. The lowest risk was seen in the third quartile of NGAL suggesting an U-shaped relationship between NGAL and mortality. Compared to conventional cardiovascular risk factors, NGAL was predictive even after adjustment for hsCRP. However, this effect was abrogated after adjustment for creatinine and eGFR. We found an inverse and highly significant nonlinear correlation of NGAL with creatinine, cystatin C and eGFR. Although eGFR appeared to associate closer with NGAL when eGFR was lower than 60mL/min/1.73m^2^ we could not prove a significant role of NGAL in patients with either reduced or normal eGFR.

Similar to our results patients with CHF secondary to CAD, but with normal renal function presented with markedly elevated plasma NGAL levels compared to individuals without CAD. Importantly, the increase in NGAL independently correlated with both the NYHA stage and the estimated GFR [30]. In our study NGAL levels were associated with diabetes and insulin resistance which is supported by other investigators [31].

As indicated by its strong correlation with each other the major amount of NGAL may be derived from neutrophils. This hypothesis is underlined by a community-based study from Copenhagen that also attributed plasma NGAL being primarily derived from neutrophils [19]. Since the main determinant of plasma NGAL are neutrophils, but monocyte-, hepatocyte-, endothelial-, smooth muscle cell-, renal tubular cells contribute also to NGAL levels the predictive role of systemic NGAL may be attenuated [2, 3].

Unfortunately, we had no urine specimens available to examine urinary NGAL. Although NGAL was significantly associated with hsCRP it provided predictive information even after inclusion of hsCRP into the regression model; NGAL may thus reflect vascular inflammation beyond CRP. Likewise, we found statistically significant associations between NGAL and cardiovascular risk factors, similar to other investigators [15, 19].

Published studies of NGAL are not entirely consistent with our results: In a Swedish study of 597 men, plasma NGAL was associated with all-cause mortality independent of other cardiovascular risk factors, but not after adjustment for CRP, cystatin C-based eGFR or albuminuria. In this study, however, urine NGAL was associated with increased all-cause and cardiovascular mortality even after adjustments for CRP and cystatin C-based glomerular filtration rate [32].

Among patients with chronic kidney disease, urine NGAL was independently associated with future ischemic atherosclerotic events, but not with heart failure events or deaths [33].

Furthermore, atherosclerosis is mediated by matrix metalloproteinases (MMPs), which are counteracted by tissue inhibitors of MMP (TIMPs) and NGAL [8, 9]. NGAL has been identified in the cytosol and in a complex containing matrix metalloproteinase-9 (MMP-9). Experimentally, vascular injury was followed by NF-kappaB-dependent expression of NGAL in vascular smooth muscle cells. NGAL obviously interacted with MMP-9, so that NGAL could modulate MMP-9 proteolytic activity in the vascular repair process [9]. Very recently it has been demonstrated that aldosterone induces MMP-9 and MMP-9/NGAL protein complex in neutrophils [13]. In general, the action of MMPs is inhibited when combined with tissue inhibitors of MMPs (TIMPs), and prolonged when these proteases bind to NGAL [34]. However, experimental data are conflicting: A significant increase in MMP9/TIMP2 complex concentration was noted in intraluminal thrombi of patients on simvastatin, but no changes in the expression of NGAL mRNA or the protein concentration of MMP9/NGAL whereas gene expression of TIMP1 in the aneurysma wall was downregulated [35].Thus, an important role of NGAL modulating inflammation and atherosclerosis is conceivable. This is underlined by a couple of publications that attributed either a predictive role to NGAL with respect to morbidity or mortality or could show NGAL expression in atherosclerotic tissue plaques and myocardial infarction [10, 15, 19, 36–42]. It is noteworthy, that lipid lowering therapy by statins stimulates the shedding of the soluble type I transmembrane protein receptor for advanced glycation end products (RAGE), and thus ameliorates atherosclerosis via acting as a decoy for RAGE ligands [43].

Of note is that our NGAL concentrations are considerably lower than those published by investigators who proposed a stronger predictive role for NGAL. However, these differences may be ascribed to different assay characteristics. Lindberg used an in-house time resolved immunofluorometric assay in the 4th Copenhagen City Heart Study, whereas we measured NGAL by using a particle-enhanced turbidimetric immunoassay [15, 19]. Nonetheless, we ought to keep in mind that the absence of an appropriate reference material to be used to calibrate the applied assays, lack of harmonization and differences in the specificity of these assays could explain the observed differences between the studies.

### Study limitations

Timing of blood samples is an issue in the studies investigating NGAL and CAD. We measured NGAL before the invasive procedure to control for influences of contrast media and increase in NGAL due to direct damage of the kidney like ischemia-reperfusion injury possibly caused by the angiography maneuver [44, 45]. Blood drawings for determination of NGAL and other markers took place once at study entry. Therefore, it is conceivable that additional blood sampling would have provided a more accurate correlation with other inflammatory markers or would have enhanced the predictive value [46]. Furthermore, besides albuminuria also urinary NGAL compared with plasma NGAL levels would have been useful to characterize its predictive value.

Since we cannot rely on a gold-standard GFR measurement we should note that even mild reductions in kidney function, putatively not reflected by creatinine or cystatin C, could modify myocardial function and consequently increase the risk for sudden cardiac death, without any clinical evidence of CHF, coronary disease, or structural changes of the myocardium. Furthermore, our study was conducted at a single tertiary referral center and may not be representative for a random population sample. Consequently, a larger number of individuals without CAD would be desirable in the LURIC cohort. However, one of the inclusion criteria of the LURIC study was the availability of a coronary angiogram and recruitment was consecutive. Since coronary angiography is done if there is a clinical suspicion of coronary artery disease, there has been an enrichment of male cases compared to females. However, angiography-based recruitment of study participants rules out that individuals with significant, yet clinically unapparent, CAD are inadvertently allocated to the control group. Further, angiographic findings represent a continuum of changes starting from wall irregularities to severe occlusion so that we used quantitative measures of coronary atherosclerosis like the Friesinger score. We are aware that optical in vivo assessment of the coronary wall with high resolution optical coherence tomography (OCT) offers a number of specific diagnostic features to study culprit lesions in patients with different clinical presentations (e.g. NSTEMI, or STEMI) and give insight into the dynamic nature of atherosclerotic plaque formation, modification and rupture. However, unfortunately, OCT analyses are not available in the LURIC cohort, since the baseline examination took place between 1997 and 2001 and the severity of CAD was assessed using coronary angiography [47, 48]. Furthermore, we have no records on non-fatal myocardial infarction or other non-fatal endpoints as the LURIC study was designed to identify biomarkers for cardiovascular and total mortality.

Because the LURIC cohort consists mainly of elderly persons, the described associations of NGAL with mortality cannot necessarily be extrapolated to younger populations even after adjusting for age. Finally, as with any observational study, there may be residual confounding of the association of NGAL with CAD, CV diseases and all-cause mortality irrespective of statistical adjustments.

## Conclusions

In the LURIC cohort plasma NGAL is independently associated with all-cause and CVD mortality even after controlling for conventional cardiovascular risk factors including hsCRP but adjustment for creatinine abrogates this effect.

## Supporting information

S1 Fig Association of eGFR, hsCRP and NGAL. A three-dimensional graph depicting the association between percentiles of hsCRP with NGAL and eGFR in which eGFR is clustered in ≤ 60 ml/min/1.73m^2^, 60–90 ml/min/1.73m^2^, and > 90 ml/min/1.73m^2^.
(PDF)

S1 Table Clinical and biochemical characteristics of study participants at baseline in individuals with and without CAD. Values are mean ± SD or median (25^th^ and 75^th^ percentile), respectively. ^a^ ANOVA or logistic regression, respectively, adjusted for age and gender. ^b^ Logistic regression, adjusted for age only. ^c^ Adjusted for use of beta blockers, ACE inhibitors, AT1 receptor antagonists, calcium channel blockers, diuretics and lipid-lowering agents. ^4^ Adjusted for use of lipid-lowering agents. ^5^ ANOVA of logarithmically transformed values.
(PDF)

S2 Table Association of NGAL with cardiovascular risk factors and coronary artery disease in individuals with and without CAD. ^a^Estimated marginal means and 95% confidence intervals obtained in a general linear model (ANOVA), adjusted for sex, gender, coronary artery disease, body mass index, diabetes mellitus, hypertension, smoking, LDL cholesterol, HDL cholesterol, triglycerides. ^b^Compared to the first category of each variable. ^c^Post hoc pairwise comparisons with the first category of each variable. ^d^Thresholds of 27 and 26 kg apply to males and females, respectively. ^e^Thresholds of 102 and 88 cm apply to males and females, respectively
(PDF)
