# Supplementary figures and images for: Neutrophil gelatinase-associated lipocalin levels are U-shaped in the Ludwigshafen Risk and Cardiovascular Health (LURIC) study—Impact for mortality
Source: PLoS One. 2017 Feb 16;12(2):e0171574. doi: 10.1371/journal.pone.0171574 (PMC5312954; doi:10.1371/journal.pone.0171574)

**Mean NGAL [ng/mL]**

120  
100  
80  
60  
40  
20  
0

**Percentile group of  
hsCRP**

**CKD EPI GFR  
Classes**

1

2

3

4

90,01+

60,01 -  
90,00

<=  
60,00

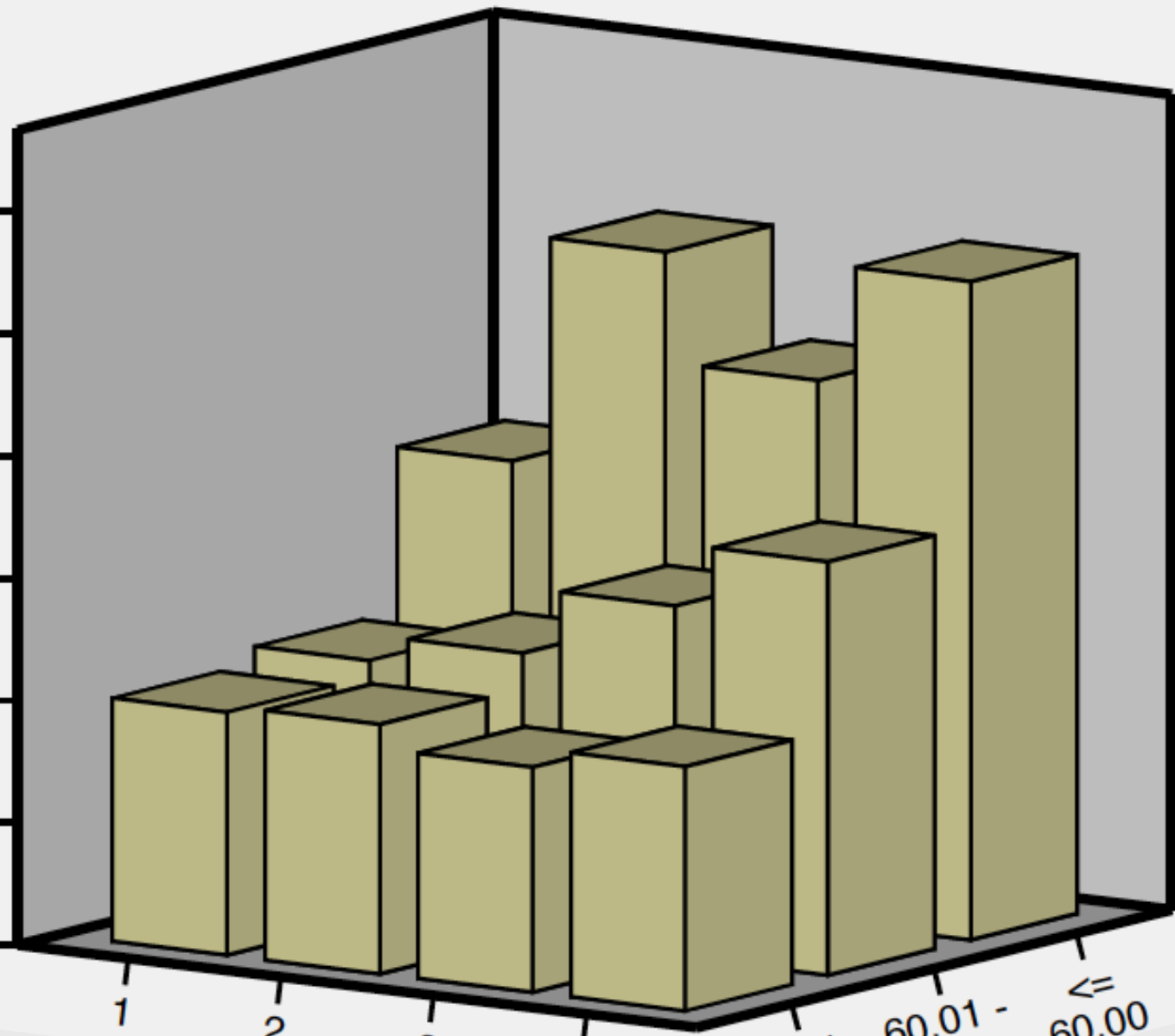

Supplement: S1 Fig — A three-dimensional graph depicting the association between percentiles of hsCRP with NGAL and eGFR in which eGFR is clustered in ≤ 60 ml/min/1.73m2, 60–90 ml/min/1.73m2, and > 90 ml/min/1.73m2. (PDF) [file pone.0171574.s001.pdf]
