# Supplementary material for: Neutrophil gelatinase-associated lipocalin levels are U-shaped in the Ludwigshafen Risk and Cardiovascular Health (LURIC) study—Impact for mortality
Source: PLoS One. 2017 Feb 16;12(2):e0171574. doi: 10.1371/journal.pone.0171574 (PMC5312954; doi:10.1371/journal.pone.0171574)
Supplement: S1 Table — Values are mean ± SD or median (25th and 75th percentile), respectively. a ANOVA or logistic regression, respectively, adjusted for age and gender. b Logistic regression, adjusted for age only. c Adjusted for use of beta blockers, ACE inhibitors, AT1 receptor antagonists, calcium channel blockers, diuretics and lipid-lowering agents. 4 Adjusted for use of lipid-lowering agents. 5 ANOVA of logarithmically transformed values. (PDF) [file pone.0171574.s002.pdf]

S1 Table

Clinical and biochemical characteristics of study participants at baseline in individuals with and without CAD.

	without CAD (n = 638)	with CAD (n = 2359)	P [a]
Age, years	59 ± 12	64 ± 10	<0.001
Male sex, %	52	75	<0.001 [b]
Body mass index, kg/m^2	27 ± 4	28 ± 4	0.683
Waist circumference, cm	97 ± 12	100 ± 12	0.062
Diabetes mellitus, %	27	44	<0.001
Insulin resistance by HOMA	2.5 ± 2.8	3.4 ± 4.1	<0.001
Systemic hypertension, %	63	76	0.008
Smoking, %			
Never	52	32	
Past	30	48	<0.001
Current	18	20	<0.001
Previous myocardial infarction, %	-	53	-
Peripheral vascular disease, %	2	12	<0.001
Cerebrovascular disease, %	5	9	0.073
Systolic blood pressure, mm Hg	136 ± 22	143 ± 24	0.004 [c]
Diastolic blood pressure, mm Hg	80 ± 11	81 ± 12	0.276 [c]
Fasting blood glucose, g/L	105 ± 28	116 ± 37	<0.001
LDL cholesterol, g/L	119 ± 31	116 ± 35	0.002 [d]
HDL cholesterol, g/L	43 ± 12	38 ± 10	<0.001 [d]
Triglycerides, g/L	133 (97-196)	150 (113-201)	<0.001[d,e]
C-reactive protein (mg/L)	2.1 (1.0 - 5.8)	3.8 (1.5 - 9.2)	<0.001[d,e]
Estimated Glomerular filtration rate, ml/min	85.9 ± 19.1	80.4 ± 20.4	0.155
Creatinine, mg/dL	0.83 ± 0.29	0.94 ± 0.55	0.037
Cystatin C, mg/L	0.92 ± 0.26	1.02 ± 0.44	0.016
NGAL, ng/ml	47.3 ± 86.5<br>39 (30 - 53)	57.1 ± 143.9<br>41 (30 - 56)	0.802

Values are mean ± SD or median (25th and 75th percentile), respectively

[a] ANOVA or logistic regression, respectively, adjusted for age and gender

[b] Logistic regression, adjusted for age only

[c] Adjusted for use of beta blockers, ACE inhibitors, AT1 receptor antagonists, calcium channel blockers, diuretics and lipid-lowering agents.

[4] Adjusted for use of lipid-lowering agents

[5] ANOVA of logarithmically transformed values.
