# Supplementary material for: Neutrophil gelatinase-associated lipocalin levels are U-shaped in the Ludwigshafen Risk and Cardiovascular Health (LURIC) study—Impact for mortality
Source: PLoS One. 2017 Feb 16;12(2):e0171574. doi: 10.1371/journal.pone.0171574 (PMC5312954; doi:10.1371/journal.pone.0171574)
Supplement: S2 Table — aEstimated marginal means and 95% confidence intervals obtained in a general linear model (ANOVA), adjusted for sex, gender, coronary artery disease, body mass index, diabetes mellitus, hypertension, smoking, LDL cholesterol, HDL cholesterol, triglycerides. bCompared to the first category of each variable. cPost hoc pairwise comparisons with the first category of each variable. dThresholds of 27 and 26 kg apply to males and females, respectively. eThresholds of 102 and 88 cm apply to males and females, respectively (PDF) [file pone.0171574.s003.pdf]

S2 Table: Association of NGAL with cardiovascular risk factors and coronary artery disease in individuals with and without CAD.

	without CAD				with CAD			
	n	NGAL (ng/mL)[a]	Difference (%)[b]	*P*[c]	n	NGAL (ng/mL)[a]	Difference (%)[b]	*P*[c]
**Gender**								
Men	320	41.3 (38.9-44.0)			1746	40.1 (38.8-41.4)		
Women	318	36.9 (34.6-39.3)	-10.9	0.016	609	41.8 (39.5-44.2	+4.1	0.235
**Age, years**								
<60	311	37.1 (34.8-39.5)			787	38.1 (36.3-40.0)		
60-70	217	40.9 (38.0-44.0)	+10.3	0.055	854	41.1 (39.3-43.0)	+7.9	0.147
>70	110	41.3 (37.2-46.0)	+11.5	0.095	717	42.6 (40.5-44.9)	+12.0	0.072
**Body mass index, kg/m^2**								
≤27 or 26[d]	303	38.7 (36.3-41.2)			1104	42.7 (41.1-44.5)		
>27 or 26[d]	335	39.4 (37.1-41.8)	1.7	0.713	1254	38.7 (37.3-40.2)	-9.4	0.001
**Waist circumference, cm**								

≤102 or 88[e]	324	40.3 (37.9-42.9)			1204	42.1 (40.5-43.7)		
>102 or 88[e]	314	37.8 (35.5-40.3)	-6.1	0.177	1154	38.9 (37.3-40.5)	-7.5	0.008
**Diabetes mellitus**								
No	465	39.9 (37.3-41.3)			1345	41.8 (40.7-43.8)		
Yes	173	38.5 (35.4-41.9)	-2.0	0.690	1013	38.4 (36.9-40.1)	-8.2	0.001
**Insulin resistance by HOMA**								
≤2.5	419	40.0 (37.9-42.3)			1284	41.0 (40.0-43.3)		
>2.5	193	37.0 (34.0-40.2)	-7.5	0.137	1016	39.1 (37.4-40.9)	-4.6	0.048
**Hypertension**								
No	228	40.0 (37.2-43.2)			587	39.7 (37.5-42.1)		
Yes	410	38.5 (36.5-40.7)	-3.8	0.435	1771	40.8 (39.5-42.1)	+2.7	0.432
**Smoking**								
Never	332	41.4 (35.2-41.3)			755	39.5 (37.5-41.6)		
Former	194	38.2 (35.2-38.1)	-7.8	0.127	1147	40.7 (39.1-42.3)	+2.9	0.392
Current	568	34.3 (30.8-38.1)	-17.2	0.003	456	42.0 (39.3-44.9)	+6.4	0.164
**Lipid-lowering drugs**								

No	518	38.9 (37.1-40.8)			1012	41.6 (38.9-43.4)		
Yes	120	39.7 (35.9-43.9)	+2.0	0.733	1346	39.7 (38.4-41.2)	-4.6	0.113
**LDL cholesterol, g/L**								
1st quartile (<0.95)		39.8 (36.1-43.9)				39.4 (37.4-41.6)		
2nd quartile (0.95-1.14)		39.6 (36.4-43.1)	-0.5	0.944		39.7 (37.6-41.9)	+0.6	0.879
3rd quartile (1.15-1.38)		37.2 (34.3-40.5)	-6.5	0.309		40.4 (38.1-42.6)	+2.3	0.602
4th quartile (≥1.39)		39.9 (36.8-43.3)	+0.2	0.974		43.0 (40.7-45.6)	+9.0	0.031
**HDL cholesterol, g/L**								
1st quartile (<0.32)		41.0 (36.4-46.1)				44.0 (41.7-46.5)		
2nd quartile (0.32-0.37)		39.9 (36.2-44.0)	-2.6	0.725		40.3 (38.2-42.4)	-8.5	0.019
3rd quartile (0.38-0.44)		38.7 (35.6-42.2)	-5.5	0.449		38.6 (36.4-40.9)	-12.5	0.001
4th quartile (≥0.45)		38.2 (35.6-40.9)	-6.9	0.325		38.7 (36.3-41.1)	-12.2	0.003
**Triglycerides, g/L**								
1st quartile (<1.09)		38.2 (35.6-41.3)				40.8 (38.4-43.3)		
2nd quartile (1.09-1.46)		41.0 (37.5-44.7)	+7.1	0.247		40.4 (38.3-42.7)	-0.8	0.841
3rd quartile (1.47-2.00)		40.5 (36.8-44.6)	+5.9	0.363		41.2 (39.1-43.5)	+1.1	0.776

4th quartile (≥2.01)		37.3 (34.0-40.8)	-2.6	0.680		39.7 (37.6-42.0)	-2.6	0.551
**C-reactive protein, mg/L**								
<3	380	37.7 (35.7-39.9)			1028	38.4 (36.8-40.1)		
3-10	180	40.4 (37.2-43.8)	+7.0	0.181	799	41.6 (39.7-43.6)	+8.3	0.014
≥10	78	42.9 (38.9-48.6)	+13.8	0.069	531	43.2 (40.6-45.9)	+12.3	0.004
**eGFR, mL/min/1.73m^2 (CKD-EPI)**								
>90	288	35.8 (33.4-38.7)			805	31.3 (29.8-32.9)		
61-90	285	39.4 (37.0-42.1)	+10.2	0.055	1197	40.4 (38.9-41.8)	+28.9	<0.001
≤60	65	55.4 (48.1-63.9)	+55.0	<0.001	353	73.0 (68.1-78.2)	+113.0	<0.001
**Creatinine, mg/dL**								
1st quartile (<0.72)	195	34.6 (31.9-37.5)				31.5 (29.6-33.5)		
2nd quartile (0.72-0.85)	173	39.1 (36.4-42.4)	+13.2	0.032		36.4 (34.5-38.4)	+15.6	<0.001
3rd quartile (0.86-0.99)	132	41.8 (38.1-45.9)	+20.8	0.005		40.6 (38.5-57.6)	+28.8	<0.001
4th quartile (≥1.00)	98	48.5 (43.4-54.5)	+40.4	<0.001		54.7 (51.9-57.6)	+73.7	<0.001
**Cystatin C, mg/L**								
1st quartile (<0.81)		34.5 (32.0-37.1)				31.3 (29.6-33.2)		

2nd quartile (0.81-0.91)		38.2 (35.3-41.2)	+10.8	0.062		34.6 (32.9-36.5)	+10.5	0.011
3rd quartile (0.92-1.06)		38.7 (35.3-42.6)	+12.4	0.060		41.0 (39.0-43.2)	+30.9	<0.001
4th quartile (≥1.07)		51.2 (46.3-56.7)	+48,6	<0.001		57.1 (54.1-60.1)	+82.0	<0.001

[a]Estimated marginal means and 95% confidence intervals obtained in a general linear model (ANOVA), adjusted for sex, gender, coronary artery disease, body mass index, diabetes mellitus, hypertension, smoking, LDL cholesterol, HDL cholesterol, triglycerides

[b]Compared to the first category of each variable

[c]Post hoc pairwise comparisons with the first category of each variable

[d]Thresholds of 27 and 26 kg apply

to males and females, respectively

[e]Thresholds of 102 and 88 cm apply to males and females, respectively
